# Volcanic arc rigidity variations illuminated by coseismic deformation of the 2011 Tohoku-oki M9

**DOI:** 10.1126/sciadv.adl4264

**Published:** 2024-06-05

**Authors:** Simone Puel, Thorsten W. Becker, Umberto Villa, Omar Ghattas, Dunyu Liu

**Affiliations:** ^1^ Department of Earth and Planetary Sciences, Jackson School of Geosciences, The University of Texas at Austin, Austin, TX 78712, USA.; ^2^ Institute for Geophysics, Jackson School of Geosciences, The University of Texas at Austin, Austin, 78758 TX, USA.; ^3^ Oden Institute for Computational Engineering and Sciences, The University of Texas at Austin, TX 78712, USA.; ^4^ Walker Department of Mechanical Engineering, The University of Texas at Austin, Austin, TX 78712, USA.

## Abstract

Rock strength has long been linked to lithospheric deformation and seismicity. However, independent constraints on the related elastic heterogeneity are missing, yet could provide key information for solid Earth dynamics. Using coseismic Global Navigation Satellite Systems (GNSS) data for the 2011 M9 Tohoku-oki earthquake in Japan, we apply an inverse method to infer elastic structure and fault slip simultaneously. We find compliant material beneath the volcanic arc and in the mantle wedge within the partial melt generation zone inferred to lie above ~100 km slab depth. We also identify low-rigidity material closer to the trench matching seismicity patterns, likely associated with accretionary wedge structure. Along with traditional seismic and electromagnetic methods, our approach opens up avenues for multiphysics inversions. Those have the potential to advance earthquake and volcano science, and in particular once expanded to InSAR type constraints, may lead to a better understanding of transient lithospheric deformation across scales.

## Teaser

Coseismic surface deformation reveals variations in elastic rock strength at depth, advancing earthquake and volcano dynamics.

## INTRODUCTION

Large-magnitude subduction zone earthquakes cause widespread deformation, including coseismic subsidence beneath volcanoes located hundreds of kilometers away from the rupture area (*1*, *2*). Associated local deformation may indicate rheological heterogeneity associated with the magmatic plumbing system (*3*, *4*) where the presence of high geothermal gradients (*3*, *5*) and crustal intrusions (*6*, *7*), for example, may mechanically weaken the rocks.

While geodetic observations have previously been used for inferences about elastic structure on global scales (*8*), here, we present the first direct inversion of coseismic crustal deformation for both upper mantle, margin-scale elastic properties and fault slip. We focus on northeastern Japan and invert coseismic surface displacements due to the 2011 Tohoku-oki M9 earthquake to investigate rigidity variations along the margin and specifically within the overriding plate’s volcanic arc. Low-seismic velocity beneath the volcanic arc in northeastern Japan have long been imaged by seismic tomography (*9*, *10*), including for individual volcanoes such as Naruko (*11*, *12*), and high *v_P_*/*v_s_* ratios at depth suggest the presence of fluids and/or partial melt (*10*, *13*). The sources of these fluids may be attributed to dehydration metamorphic reactions of minerals from the subducting slab at ~100 km depth, above which many of the volcanic arcs worldwide are typically located (*14*, *15*).

Beneath the volcanoes and inland northeastern Japan, the upper-plate structure exhibits shallow seismicity within the brittle deformation regime. In addition, deeper low-frequency earthquakes occur around active volcanoes, often associated with magmatic activity (*9*). In contrast, seismic activity is notably elevated and more widespread offshore, in particular along the primary thrust region beneath the landward slope of the Japan Trench. Several studies involving seismic, gravity, and residual topography analyses have linked the segmentation of megathrust events to material heterogeneities in the overriding plate (*16*, *17*), suggesting a substantial influence of structural heterogeneities on earthquake nucleation and rupture style (*18*). For instance, Bassett *et al*. (*16*) proposed that variations in the forearc lithology of the upper plate played a key role in the along-strike variations in the 2011 Tohoku-oki M9 earthquake slip distribution. It is thus important to independently quantify the extent and distribution of the Earth’s rigidity structure within a fault and volcano dynamics context.

## RESULTS

### Joint inversion of geodetic data

**Fig. 1.**
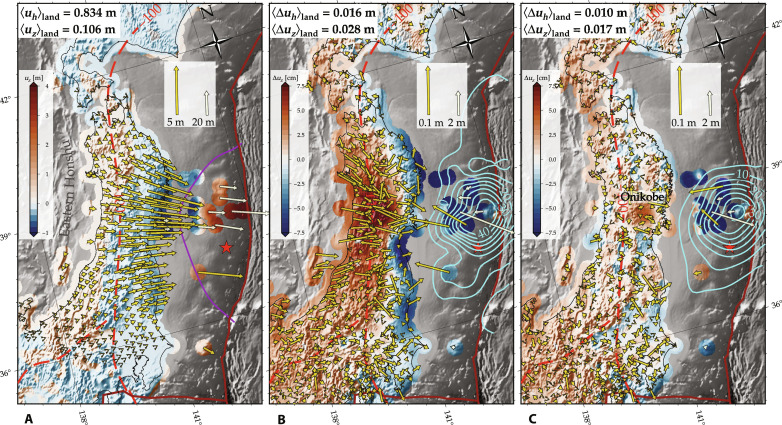
Coseismic geodetic data, displacement residuals, and inferred fault slip distribution for homogeneous and heterogeneous structure. (**A**) Coseismic deformation of the 2011 Tohoku-oki M9 earthquake (*20*–*22*). Yellow and white arrows indicate horizontal displacement vectors on different scales and uplift is represented by the background color. The red star is the Tohoku-oki epicenter, and the purple contour represents the 5-m slip contour from an earlier coseismic slip inversion (*24*). The dark red lines are major plate boundaries (*61*), and the red dashed line depicts the 100-km contour line of the subducting Pacific slab (*62*). (**B** and **C**) Displacement residuals (on different scale) and fault slip (5 m cyan contours) from our homogeneous medium slip inversion (B), and our joint inversion for fault slip and 3D shear modulus variations (C), as shown in Fig. 2. Mean values in averaging brackets indicate the root mean square (RMS) values (A) and residuals [(B) and (C)] of horizontal (***u***_***h***_) and vertical (***u***_***z***_) displacements, restricted to the map area on land.

We seek to infer variations in elastic parameters directly from coseismic geodetic deformation observations (*19*) associated with the 2011 Tohoku-oki M9. We use three-dimensional displacement information from 1283 permanent Global Positioning System (GPS) sensors, eight seafloor acoustic GPS sensors (GPS/A), and six pressure gauges (APG); the latter measure verticals only (*20*–*22*). The M9 resulted in major deformation (*20*–*22*), with ∼5-m displacements recorded horizontally in the eastern part of the Tohoku region on land, and over 30 m on the seafloor (Fig. 1A) with data corrected to remove aftershock effects (*23*, *24*). Vertical deformation showed ∼5 m of uplift near the Japan Trench and ∼1 m of subsidence near the Pacific coast of Honshu. Observational errors for the inland and offshore stations are inferred to range from a few millimeters to several centimeters (table S1).

We perform an inversion of the geodetic data; first for fault slip alone, and then for slip and three-dimensional (3D) rigidity variations (Materials and Methods). Our model domain covers a 3700 × 4600 km rectangular region around Honshu with a depth of 700 km. We refine the mesh around the epicenter and the central part of eastern Honshu (fig. S1), where we anticipate the highest information recovery from the geodetic data (Materials and Methods). Figure 1B shows the inferred fault slip and residuals between model predictions and horizontal and vertical coseismic deformation of our best homogeneous structure model. The misfit is at the ≈2 and ≈26% level for the horizontals and verticals on land, respectively (Fig. 1B). The horizontal and, more so, the vertical residuals are clearly not random but show spatially coherent patterns. Such residuals are also apparent in earlier homogeneous medium inversions (*25*). Their origin in terms of a contrast between strong slab and weak overriding plate and/or mantle has previously been explored by forward computations exploring the role of elastic heterogeneity (*24*–*27*).

**Fig. 2.**
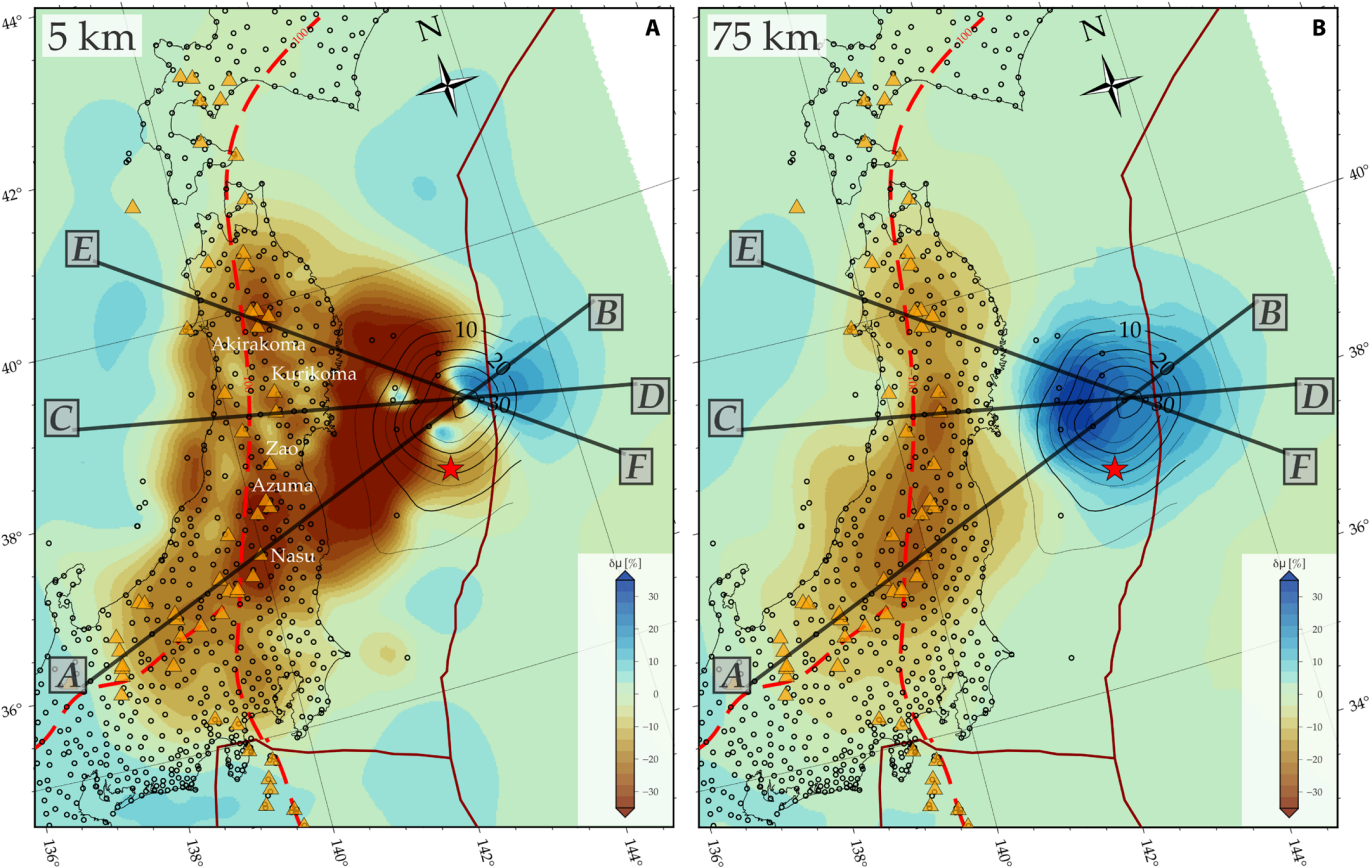
Map view of rigidity variations from the 3D joint inversion. The shear modulus, or rigidity, variations (δμ) are shown relative to the background in percent for our preferred joint inversion (Fig. 1C) at depths of 5 km (**A**) and 75 km (**B**). Geodetic sites are marked with black circles, volcanoes (*63*) are denoted by orange triangles. Slip contours in 5-m intervals are represented with black contours. Plate boundaries, slab contours, and epicenter as in Fig. 1. Profiles indicated are shown in Fig. 3.

**Fig. 3.**
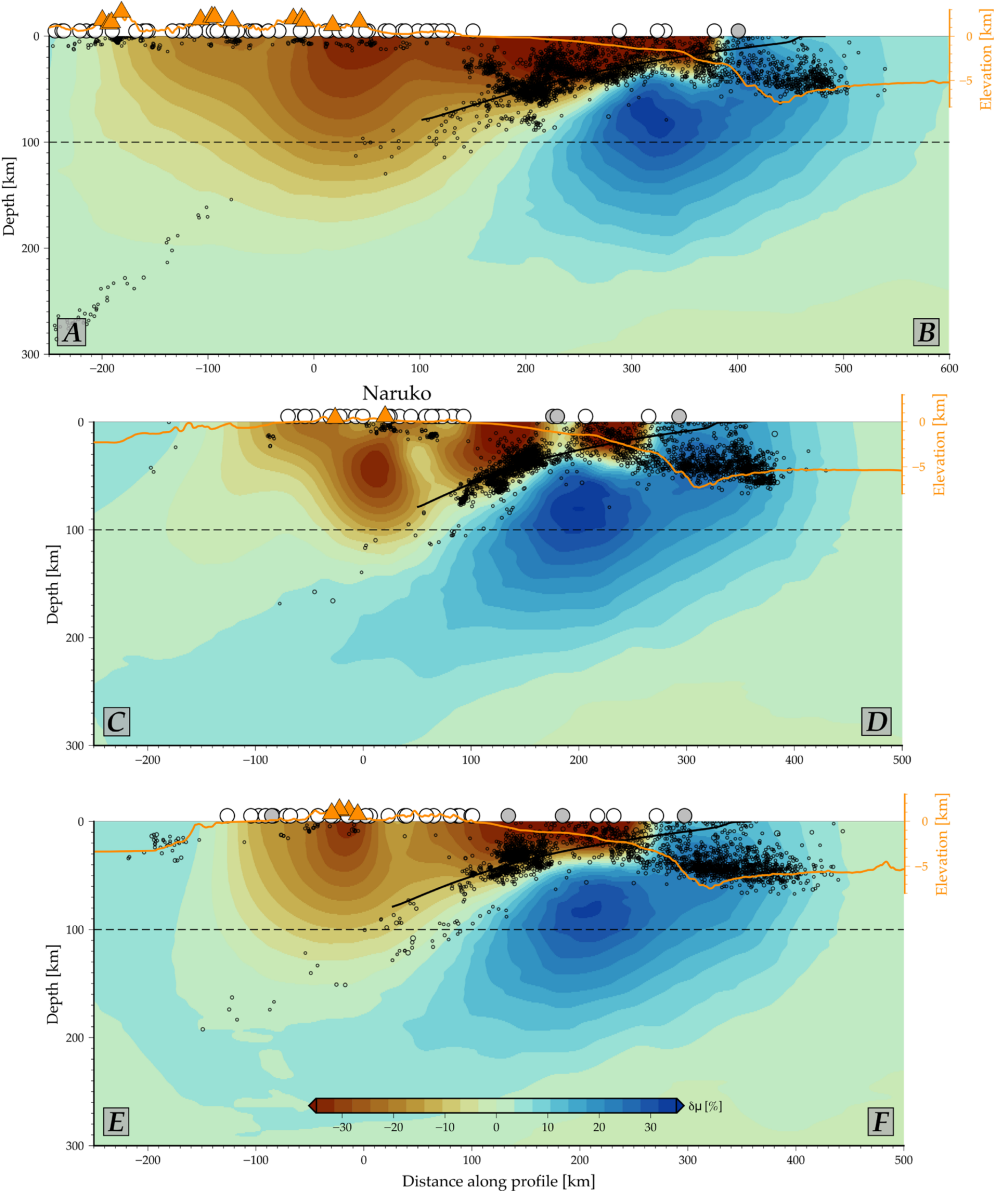
Vertical profiles illustrating rigidity (shear modulus) variations (δμ) obtained from the 3D joint inversion. The corresponding profile locations are shown on Fig. 2. The orange line represents the topography and bathymetry, while the orange triangles indicate the locations of volcanoes (*63*). White and gray dots indicate the location of the GPS stations and the APG along the profiles, respectively. The black points correspond to seismicity with a *M*_w_ > 3.5 from the Japan Meteorological Agency Catalogue between 1973 and 2023. The black solid and dashed lines depict the geometry of the Pacific slab and the 100-km depth line, respectively. This depth region represents the level at which the release and upward migration of hydrous minerals’ metamorphic reactions within the subducting slab are considered to occur, contributing to the formation of the volcanic arc.

Figure 1C shows that the residuals can be markedly reduced if we allow for 3D rigidity variations during a joint inversion for fault slip and rigidity, with the corresponding shear modulus variations shown in Figs. 2 and 3. In this case, root mean square (RMS) misfit values between model and geodetic data on land are roughly halved, to ≈1 and ≈16% for the horizontals and verticals, respectively (Fig. 1C and table S1). By jointly inverting for rigidity variations and coseismic slip, the remaining misfit patterns now lack any obvious, overall coherent structure. Only a small region in the eastern part of the Onikobe volcanic area shows overestimated vertical displacements.

The residual reduction is possible because deep elastic modulus variations have subtle but robust diagnostic surface deformation signatures for a given slip geometry (*19*, *27*), and the data appear to resolve an overriding plate that is weaker than the slab and additional anomalies in the mantle wedge that are associated with the volcanic arc (Fig. 2). Comparing the slip distribution inferred assuming a homogeneous structure with the joint inversion (contours in Figs. 1, B and C, and colored figs. S2, A and B), we find similar slip distributions with most slip concentrated near the trench, consistent with previous inversions with prescribed 3D shear modulus variations (*24*, *26*). The preferred joint inversion estimates a slightly lower maximum slip of 41 m compared to 46 m in the homogeneous case and has relatively smoother slip distribution, highlighting the trade-offs with fault-proximal structure. This reduction in slip magnitude broadly aligns with previous studies considering heterogeneous material structure in forward tests (*24*, *26*, *27*). Higher slip near the trench might be likely, e.g., from bathymetric surveys (*28*), within uncertainties (*29*, *30*), for example, and additional constraints could be included in future slip inversions. In our tests, we also find a moderate trade-off of maximum slip with slab geometry (fault dip; figs. S2C and S8), whereas the 3D rigidity structure is quite stable with respect to fault dip (figs. S6 and S7).

### Rigidity structure and weaker volcanic arc

Concentrations of more compliant material underneath the Japanese landmass correlate well with the location of volcanic centers between 37° and 42°N (Fig. 2) where GPS spatial coverage and resolution are high (fig. S3). On the basis of a number of synthetic tests, we found these features to be robust outcome of the inversion (e.g., figs. S4 and S5). At shallow depths (Fig. 2A), rigidity reductions of ∼15 to 25% are observed around Mt. Akitakoma (40°N), Mt. Kurikoma (39°N), Mt. Zao (38°N), and reductions between 25 and 35% are seen beneath Mt. Azuma (∼37.5°N) and Mt. Nasu (37°N). These volcanoes experienced notable subsidence during the 2011 Tohoku-oki event (*1*).

**Fig. 4.**
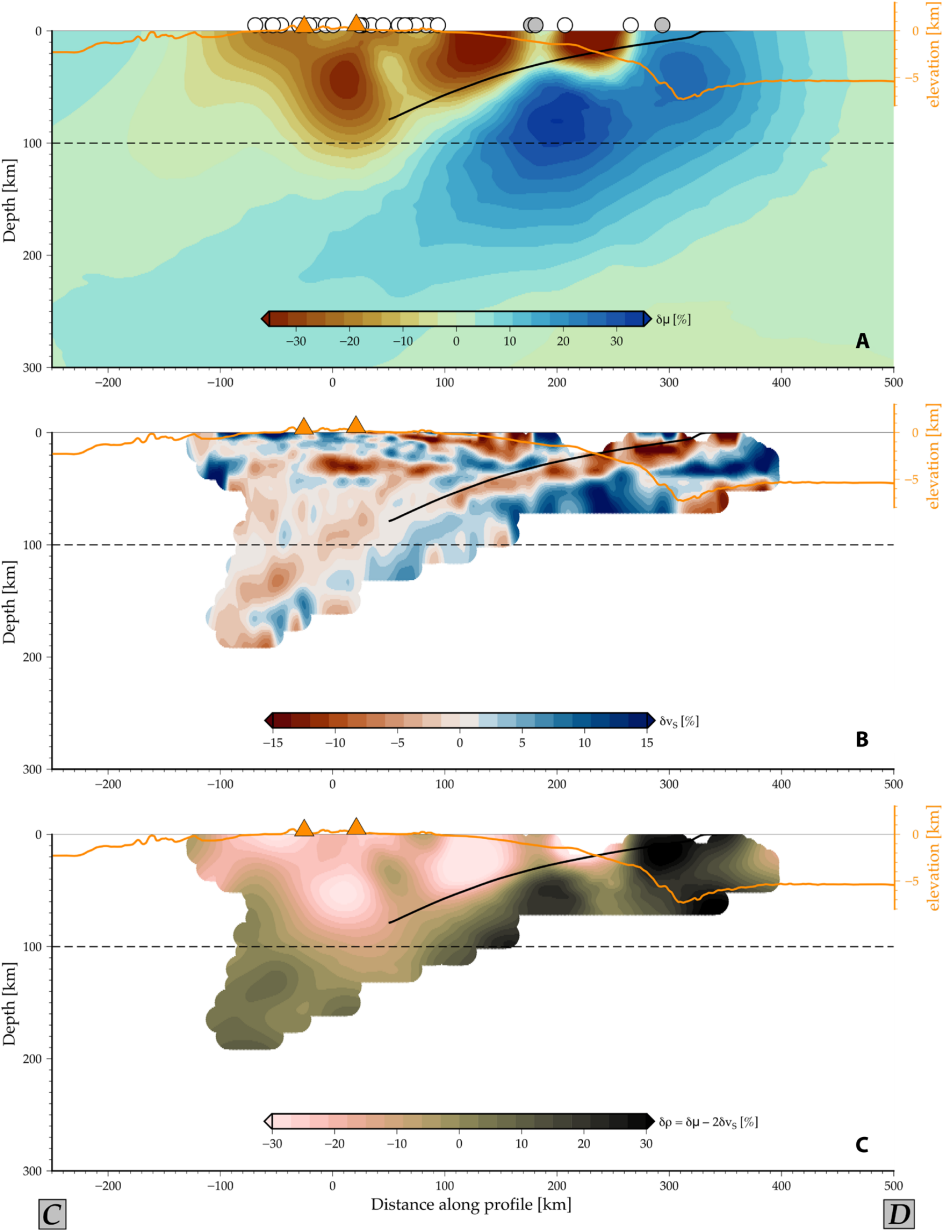
Comparison between rigidity and seismic velocity variations and density inference. (**A** and **B**) Rigidity variations (δμ) from the 3D joint inversion (A; Fig. 3) and seismic velocity variations (*33*) (δ*v_s_*; B) of vertical C-D profile (Fig. 2). (**C**) Inference of density anomalies (δρ) calculated from rigidity variations and a smoothed version of the seismic tomographic model (B). Topographic elevation, slab geometry, volcano, and station locations as in Fig. 3.

The presence of high geothermal gradients (*3*, *5*), and presence of deep-seated hot plutonic bodies (*6*) in this area aligns with the rigidity reduction due to increased temperatures weakening the rocks. These weaker material anomalies are prominent within the uppermost 30 km, which corresponds to the average crustal thickness in northeastern Japan (*31*) and to the region of reported shallow seismicity (black dots in Fig. 3), some of which may be associated with the volcanic plumbing systems. Seismic tomography of the Japan arc (*32*, *33*) suggests a correlation between the more compliant anomalies we image and low seismic velocity anomalies at shallow depths (Fig. 4).

An interesting observation in profile C-D of Fig. 3 is a lower rigidity region with a ~30% shear modulus reduction between 30 and 50 km depth beneath Naruko volcano, surrounded by a slightly stronger region (reduction below 20%). This deeper anomaly is well correlated with low-velocity anomalies and high *v_P_*/*v_s_* ratios observed beneath this volcano at these depths (*11*, *12*, *34*), possibly indicating the presence of a deeper magma reservoir. This deeper magma storage beneath Naruko, as well as shallower reservoirs beneath other volcanoes, may be directly connected to the source region of melt and fluids associated with metamorphic reactions within the subducting slab (*35*).

Notably, the volcanic low-rigidity anomalies in Figs. 2B and 3 align with the subducting slab interface reaching ~100 km depth, and the equivalent distance from the trench is typically associated with the main volcanic arc (*14*, *15*). Shear modulus reductions between 8 and 12% are apparent at 75 km depth (Fig. 2) along the volcanic arc, suggesting the possible presence of fluids and/or melt at these depths. Mechanisms involving mantle wedge dynamics and grain size effects may contribute to melt generation and magma migration from these reservoirs to the volcanic fronts (*15*, *36*). While our model lacks the vertical resolution to speculate on the migration paths of these magmatic systems from the melt reservoir to the volcano, a checkerboard resolution test suggests that the lateral resolution remains good up to approximately 50 to 60 km depth (fig. S3).

Offshore (negative elevation profiles in Fig. 3), the low-rigidity material of the upper-plate contrasts with the mechanically stronger Pacific slab (blue colors in Fig. 2B and 3). The clear distinction between these two separate domains is confirmed by synthetic tests (figs. S4 and S5), and this feature is responsible for the reduction of geodetic residuals offshore compared to the homogeneous model (Fig. 1), with remaining misfit mainly within uncertainties, except for station GTJ4 (table S1). The imaged large-scale variations in shear modulus substantiate the inferences based on forward tests (*24*, *26*).

Our model’s resolution is limited offshore due to the station coverage and trade-offs between the joint parameters (figs. S3 and S5) (*19*). However, general patterns are confirmed even if the geometry of the Pacific plate’s slab interface is not accurately known (figs. S6 and S7). The more compliant forearc structure, with rigidity reductions exceeding 30%, is well correlated with low seismic velocity anomalies in this area (*37*) and the region of highest seismicity (black dots in Fig. 3). This may indicate mechanically weaker accretionary materials and sediments containing abundant fluids (*38*), such as mudstones and pelagic clay recovered from ocean drilling expeditions (JFAST) in the frontal prism after the 2011 earthquake (*39*).

An intriguing observation from Fig. 3, profile C-D, is the segmentation of the seismic activity, as well as the weaker materials, in the forearc. This relatively higher rigidity channel appears to coincide with a region of low seismicity, possibly indicating the control of the upper-plate structure on seismicity patterns. Overall, our results suggest that while geodetic inversions yield consistent estimates of fault slip distributions only on large spatial scales (*18*), this smoothness does not imply that all information contained in coseismic surface displacements is exploited by standard elastic modeling. Additional insights into Earth’s structure and dynamics can be gained by inverting for the medium surrounding the fault.

## DISCUSSION

### Implications of geodetic constraints for material properties

Seismic tomography is commonly used to infer lithospheric and mantle structure in subduction zones based on wave speed variations. These anomalies can be further converted into, e.g., temperature or density variations based on a range of assumptions. Shear-wave velocities depend on the square root of the shear modulus divided by density; for instance, the ~30% rigidity reduction beneath the volcanoes that our model recovers (Fig. 4A) corresponds to ≈16% shear-wave reduction for constant density. Variations up to that order are found in the shear-wave anomalies in regional tomographic inversions (Fig. 4B), recognizing that both tomography and rigidity inversions are subject to damping, which leads to underestimations of anomaly strength, and spatially variable coverage and hence resolution.

Our method stands apart from traditional seismic inversions because it offers direct inferences of shear modulus derived from geodetic data, which adds further constraints on the properties of interest. For example, combining our joint inversion with tomographic models in well-constrained regions may allow us to infer density variations more robustly. The density estimate of Fig. 4C is based on combining our inferred 3D rigidity and a smoothed version of shear-wave tomography (*33*), where smoothing is required to achieve comparable representations since seismic models provide finer-scale features than our geodetic inference.

Negative density anomalies in Fig. 4C appear primarily associated with sediments and weaker rocks offshore, as well as regions where we expect magmatic reservoirs beneath the volcanoes. Positive density anomalies are found in the subducting slab, as expected. The robustness of second-order variations could be further explored in conjunction with gravity constraints (*16*). More generally, integrating different geophysical information holds the potential to yield more robust estimates of other dynamically relevant parameters for subduction zone dynamics, including temperature and the degree of partial melting.

The complex and highly heterogeneous elastic structure in northeastern Japan we imaged thus complements information from seismic tomography (*17*, *33*, *40*), while providing better constraints on coseismic fault behavior (*24*, *27*, *41*–*43*). This also emphasizes the critical role of material heterogeneity in fault slip inversions and potentially inferring fault stress state, in particular in the presence of a lower-rigidity volcanic arc and upper plate, as well as a stronger slab (*24*, *26*, *27*). Such material property anomalies also appear associated with a heterogeneous viscoelastic, postseismic response where low viscosity beneath the volcanic arc has been invoked to account for subsidence around Quaternary volcanoes (*44*–*46*).

Our joint inversion for 3D elastic variations provides insights into lithospheric dynamics beneath northeastern Japan. We identified low-rigidity material beneath volcanoes, offering a new tool, e.g., for potentially imaging time-dependent magmatic systems. More generally, our approach complements other geophysical inversion techniques. Rigidity imaging has the potential to be integrated with seismic and electromagnetic inversions, enabling a comprehensive and mechanically consistent characterization of thermo-mechanical subduction zone structure, for example. Our method promises to be especially powerful in settings where densely sampling InSAR deformation time series are available, suggesting the potential for new ways of constraining fault and volcano dynamics.

## MATERIALS AND METHODS

### Cosesmic displacement data

We use the coseismic deformation data compilation of Hashima *et al*. (*24*), which consists of 1283 terrestrial geodetic stations managed by the Geospatial Information Authority of Japan. This agency regularly releases daily site location solutions estimated through routine analysis. The effects of the aftershocks were removed following Nishimura *et al*. (*23*). In addition to the onshore sensors, we incorporated data from eight acoustic GPS sensors (GPS/A) and six pressure gauges. Among the eight acoustic GPS sensors, six were deployed by the Japan Coast Guard, while the remaining two were installed by Tohoku University (*20*, *21*). Tohoku University also deployed four out of the six pressure gauges (*22*, *47*), whereas the other two were deployed by the Earthquake Research Institute of the University of Tokyo (*48*).

One of the GPS/A stations, GJT3, recorded vertical displacements using both the acoustic GPS and a pressure gauge. We opted to use the pressure gauge data for vertical displacement, considering its higher accuracy compared to the GPS/A measurements (*24*). The offshore stations exhibit larger errors compared to the onshore data (see table S1) (*20*, *24*, *25*). The GPS and APG sensors provide continuous data, while GPS/A observations are from campaign mode measurements (*25*). Besides primarily recording the Tohoku-oki M9 coseismic displacement, the GPS/A data collected soon but not immediately after the M9, may therefore also contain signals from foreshocks, aftershocks, and postseismic effects (*20*, *21*). However, the postseismic contributions should be relatively small (a few tens of centimeters) and thus likely within uncertainties (*20*, *21*). Thus, while our results may be affected by undersampled transients like other coseismic study using these data, results are unlikely to be notably biased in this sense (see fig. S3).

### Model setup

We constructed a 3D Cartesian model of Japan, which incorporates the Pacific plate boundary and the subducting slab. The fault interface geometry was derived based on interplate seismicity, following the approach outlined in Hashima *et al*. (*24*), to allow for comparison. We do not incorporate the Philippine Sea plate and the Nankai Though, as previous studies have demonstrated their minimal impact on the overall coseismic slip distribution during the Tohoku-oki earthquake (*24*).

We converted the spherical coordinates to Cartesian coordinates using an azimuthal equidistant projection with a fixed center point at (140°E, 40°N). For simplicity, we also use a flat surface, disregarding both topography and bathymetry effects. Neglecting sphericity typically induces small changes in surface displacements (*49*). These errors, comparable to those observed in offshore noisy data (table S1), are not expected to notably alter rigidity variation patterns, although minor amplitude errors may result. Neglecting the effects of topography could potentially lead to bias in slip inversions (*50*, *51*), and we expect such effects to be most important offshore where bathymetry changes more strongly than topography on land. Offshore, our model has poor resolution due to the station coverage (fig. S3) and bathymetric gradients might further obscure rigidity structure beyond the trade-offs with fault slip (*19*). We intend to explore such more example trade-offs in future Bayesian inversions.

On the basis of the fault interface geometry, we generated a 3D mesh using the open-source mesh generation software GMSH (*52*). The mesh was refined around the Tohoku-oki earthquake epicenter and the central part of eastern Honshu (fig. S1), where we expect most of the information that can be recovered from the surface geodetic data (fig. S3). Our model domain is divided into ≈150,000 tetrahedral elements. The characteristic length of tetrahedra in the mesh ranges from ~10 km near the trench to ~200 km near the lateral and bottom sides of the model domain. For the region of interest, we kept the smallest elements of 10 km fixed up to ~100 km depth to ensure good vertical resolution, gradually increasing toward the bottom of the domain. We have verified that further mesh refinement only yielded negligible changes in the predicted displacement field.

Specifically focusing on the fault, we applied additional refinement near the earthquake location and the areas where we anticipated the highest information recovery concerning slip and subduction zone structure. In these regions, the smallest triangular element on the fault interface had a length of 10 km, gradually increasing to approximately 40 km when moving away from the targeted region. We also confirmed that this choice of reducing the fault mesh resolution outside the expected Tohoku-oki earthquake slip area had no impact on our inverse results.

### Forward problem

To solve the elastic forward problem, we follow Puel *et al*. (*53*) using a mixed finite element approach that incorporates a fault discontinuity (*53*), with an implementation based on the open-source FENICS library (*54*). Our forward model was discretized using a second-order stable triplet of finite-element spaces (*19*, *53*), resulting in ≈12.5 million degrees of freedom for the primary variables, namely, stress, displacement, and rotation. For all our tests, we imposed zero displacements on the lateral and bottom boundaries and verified that these boundaries are far enough from the region of interest to affect the model results.

To represent the slip vector ***s*** = (*s*_strike_, *s*_dip_) on the fault interface, we used the fault local coordinate system instead of expressing the components in the global model coordinates. We restricted the slip to occur only in the strike and dip directions, not allowing opening in the perpendicular direction to the fault plane. To write the slip in its strike and dip directions, we need to substitute the fault term in the elasticity variational form in Puel *et al*. (*53*) with

$$\int_{\Gamma_{F}}\left[T^{\text{strike}}\left(n_{\Gamma_{F}}\right)s_{\text{strike}}+T^{\text{dip}}\left(n_{\Gamma_{F}}\right)s_{\text{dip}}\right]\cdot \left(\tau n_{\Gamma_{F}}\right)dS$$

where Γ_F_ is the fault internal boundary. $T^{\text{strike}}\left(n\right)=\frac{n\times z}{\sqrt{\left(n\times z\right)\left(n\times z\right)}}$ and *T*^dip^(***n***) = ***n*** × *T*^strike^(***n***). ***n*** × ***z*** indicates the cross product between the unit normal vector to the fault plane and the vertical basis ***z*** = (0,0,1). **τ** is a test function, while ***n***_Γ_F__ indicates the unit normal to the fault plane, and *d****S*** is the integration over the fault boundary. We used the sparse direct solver MUMPS to solve both the forward and inverse problems.

### Coseismic slip inversion

We used the open-source library HIPPYLIB (*55*, *56*) for all inversions. Gradient and Hessian information of the least-squares cost functional that penalizes a combination of data misfit and model roughness are computed using adjoint-based optimization methods (*57*).

The fault slip inversion was performed without computing Green’s functions following Puel *et al*. (*53*). We used a Tikhonov regularization that include a weighted sum of the squared *L*^2^ norm of slip gradient with penalty term γ and the squared *L*^2^ norm of the slip itself with penalty term δ. The first term promotes smoothness of the fault split solution, and the second term ensures strong convexity of the regularization term. To determine suitable penalty weights, we fixed the ratio γ/δ at 10^9^ to allow variations of slip of the order of ~30 km. Subsequently, we performed an L-curve analysis (*58*) to identify the preferred value of γ, which was found to be ∼100.

We scaled the data misfit by the data noise covariance given the observational errors in table S1. We assigned different weights to the horizontal and vertical components of the displacement field to reproduce seafloor deformation, as suggested by several studies (*24*, *25*, *59*). To test the effect of these weights, we performed several coseismic slip inversions assuming a homogeneous medium, computing the *L*^2^ norm of the residual, weighted by the noise uncertainties. By examining the intersection of the different curves, we selected the weighting ratio that minimized this metric. The best ratio was found to be $1:\frac{1}{2}:\frac{1}{13}:\frac{1}{10}$ for horizontal components of the onshore data, vertical components of the onshore data, seafloor acoustic data, and pressure gauges vertical displacements, respectively (fig. S9).

Using these weighted data, we first performed a coseismic slip inversion for a homogeneous, elastic medium using all available data to determine the fault slip distribution during the Tohoku-oki earthquake, assuming a homogeneous medium with Poisson ratio of 0.25. The slip was discretized using linear Lagrange elements, resulting in approximately 3000 degrees of freedom for each component (strike and dip). We used a conjugate gradient (CG) algorithm preconditioned by the regularization to minimize the gradient (*53*). The inverse solution converged in ~400 CG iterations, reducing the gradient norm by 12 orders of magnitude.

### Joint inversion

The joint inversion method is built upon the approach proposed by Puel *et al*. (*19*). For consistency, we used an identical 3D mesh and data weighting as used in the homogeneous coseismic slip problem. Rigidity, represented by the shear modulus parameter of linear elasticity, was determined using a hyperbolic tangent function parameterization to ensure nonnegativity. The resolved patterns remain insensitive to this choice (*19*). Without stress constraints, our inversion is only sensitive to relative rigidity variations; we constrained the shear modulus to vary between ±50% from a nominal shear modulus background of 60 GPa, while keeping a constant Poisson’s ratio of 0.25.

To regularize both the coseismic slip and the shear modulus structure beneath Japan, we used Tikhonov regularization. The penalty weights for the slip components remained the same as in the homogeneous case, while for the shear modulus parameter, we considered a correlation length ( $\sqrt{\gamma /\delta }$ ) of ≈30 km, given that the average spacing between land stations is ~20 to 25 km. The value of γ used in our inversion was again determined from L-curve analysis, resulting in a value of 400 (fig. S10). The shear modulus structure was discretized using linear Lagrange elements resulting in ≈25,000 degrees-of-freedom. We used an inexact Newton-CD algorithm (*60*) to solve the optimization problem, and convergence was achieved within ~20 iterations, reducing the norm of the gradient by six orders of magnitude.

To assess the robustness of our results, we conducted three synthetic recovery and several checkerboard tests. The first one consisted of 3D synthetics where we prescribed the inferred slip from the homogeneous slip inversion and introduced a subduction zone structure with various rigidity values, including a higher rigidity subducting slab (75 GPa), a weaker overriding plate (45 GPa), and lower rigidity spherical anomalies (35 GPa) beneath the volcanic arc within a 30 km radius (fig. S4). We polluted the synthetic data with random Gaussian noise with SD of 1.5 cm. Results show successful recovery of the stronger slab and weaker overriding plate except close to the trench, likely due to trade-offs between slip and material heterogeneity (*19*). Moreover, our model accurately captured the weaker material beneath the volcanoes in the region of interest up to depths of ≈50 to 60 km.

The effects of the low spatial coverage offshore and trade-offs between the inferred slip and material heterogeneity are more pronounced in a second, antithetical test in which the heterogeneous structure is characterized by a hypothetical weaker subducting slab (35 GPa) and a stronger upper-plate (75 GPa) and volcanic arc (85 GPa) (fig. S5). Although the results show an overall good recovery of the higher rigidity in the overriding plate and the weaker subducting slab in the region of highest resolution (fig. S3), the joint inversion seems to prefer the recovery of a weaker forearc and a stronger slab near the trench.

The third test repeated our reference joint inversion but increased the Pacific plate dip by 10° to evaluate the robustness of the results when the fault geometry is uncertain. The results from this test in figs. S6 and S7 show similar rigidity results as in our reference case (Figs. 2 and 3) with only minor changes in the subduction zone structure and slightly higher slip toward the trench (fig. S2C).

Model resolution was evaluated through a checkerboard test (fig. S3). The test used a sinusoidal model perturbation with a maximum shear modulus variation amplitude of ±35% in a 50 km × 50 km × 50 km grid. On the basis of the recovery, our model provides regional constraints onshore above depths of 40 to 50 km, with good resolution extending to 50 to 60 km beneath the volcanoes. Offshore, resolution is poor due to limited station coverage, with the highest resolution occurring at the intersection point of the profiles (143°E, 38°E) up to depths of 30 to 40 km.

### Density calculation

To estimate the density variations from rigidity and shear-wave velocity variations, we take the derivative of the *v_s_* formula, $v_{s}=\sqrt{\mu /\rho }$ , with respect to both shear modulus and density. Expressed as relative variations, the relationship reads: δρ = δμ − 2δ*v_s_*.

## References

[1] Y. Takada, Y. Fukushima, Volcanic subsidence triggered by the 2011 Tohoku earthquake in Japan. Nat. Geosci. 6, 637–641 (2013).

[2] M. E. Pritchard, J. A. Jay, F. Aron, S. T. Henderson, L. E. Lara, Subsidence at southern Andes volcanoes induced by the 2010 Maule, Chile earthquake. Nat. Geosci. 6, 632–636 (2013).

[3] T. Yoshida, The evolution of arc magmatism in the NE Honshu arc Japan. Tohoku Geophys. J. 36, 131–149 (2001).

[4] T. Sagiya, A. Meneses-Gutierrez, Geodetic and geological deformation of the island arc in northeast Japan revealed by the 2011 Tohoku earthquake. Annu. Rev. Earth Planet. Sci. 50, 345–368 (2022).

[5] G. Sangyō, K. Sōgō, C. S. S. Chishitsu, Distribution map and catalogue of hot and mineral springs in Japan. In Japanese with English abstract., 2nd edn, Digital Geosci. Map GT-2, Natl Inst. of Adv. Ind. Sci. and Technol. *Geological Survey of Japan*, Tsukuba, Japan (2005).

[6] N. Doi, O. Kato, K. Ikeuchi, R. Komatsu, S. Miyazaki, K. Akaku, T. Uchida, Genesis of the plutonic-hydrothermal system around Quaternary granite in the Kakkonda geothermal system, Japan. Geothermics 27, 663–690 (1998).

[7] A. Tanaka, M. Yamano, Y. Yano, M. Sasada, Geothermal gradient and heat flow data in and around Japan (I): Appraisal of heat flow from geothermal gradient data. Earth Planets Space 56, 1191–1194 (2004).

[8] H. C. P. Lau, J. X. Mitrovica, J. L. Davis, J. Tromp, H. Y. Yang, D. Al-Attar, Tidal tomography constrains Earth’s deep-mantle buoyancy. Nature 551, 321–326 (2017). 29144451 10.1038/nature24452

[9] A. Hasegawa, D. Zhao, S. Hori, A. Yamamoto, S. Horiuchi, Deep structure of the northeastern Japan arc and its relationship to seismic and volcanic activity. Nature 352, 683–689 (1991).

[10] D. Zhao, A. Hasegawa, S. Horiuchi, Tomographic imaging of P and S wave velocity structure beneath northeastern Japan. J. Geophys. Res. Sol Earth 97, 19909–19928 (1992).

[11] J. Nakajima, A. Hasegawa, Tomographic imaging of seismic velocity structure in and around the Onikobe volcanic area, northeastern Japan: Implications for fluid distribution. J. Volcanol. Geotherm. Res. 127, 1–18 (2003).

[12] T. Okada, T. Matsuzawa, J. Nakajima, N. Uchida, M. Yamamoto, S. Hori, T. Kono, T. Nakayama, S. Hirahara, A. Hasegawa, Seismic velocity structure in and around the Naruko volcano, NE Japan, and its implications for volcanic and seismic activities. Earth Planets Space 66, 114 (2014).

[13] J. Nakajima, T. Matsuzawa, A. Hasegawa, D. Zhao, Seismic imaging of arc magma and fluids under the central part of northeastern Japan. Tectonophysics 341, 1–17 (2001).

[14] Y. Tatsumi, S. Eggins, *Subduction Zone Magmatism* (Blackwell, Oxford, 1995), vol. 1.

[15] P. C. England, R. F. Katz, Melting above the anhydrous solidus controls the location of volcanic arcs. Nature 467, 700–703 (2010). 20930842 10.1038/nature09417

[16] D. Bassett, D. T. Sandwell, Y. Fialko, A. B. Watts, Upper-plate controls on coseismic slip in the 2011 magnitude 9.0 Tohoku-oki earthquake. Nature 531, 92–96 (2016). 26935698 10.1038/nature16945

[17] X. Liu, D. Zhao, Upper and lower plate controls on the great 2011 Tohoku-oki earthquake. Sci. Adv. 4, 4396 (2018). 10.1126/sciadv.aat4396 PMC6010320 29938226

[18] N. Uchida, R. Bürgmann, A decade of lessons learned from the 2011 Tohoku-Oki earthquake. Rev. Geophys. 59, e2020RG000713 (2021).

[19] S. Puel, T. W. Becker, U. Villa, O. Ghattas, D. Liu, An adjoint-based optimization method for jointly inverting heterogeneous material properties and fault slip from earthquake surface deformation data. Geophys. J. Int. 236, 778–797 (2024).

[20] M. Sato, T. Ishikawa, N. Ujihara, S. Yoshida, M. Fujita, M. Mochizuki, A. Asada, Displacement above the hypocenter of the 2011 Tohoku-Oki earthquake. Science 332, 1395–1395 (2011). 21596950 10.1126/science.1207401

[21] M. Kido, Y. Osada, H. Fujimoto, R. Hino, Y. Ito, Trench-normal variation in observed seafloor displacements associated with the 2011 Tohoku-Oki earthquake. Geophys. Res. Lett. 38, 2011GL050057 (2011).

[22] Y. Ito, T. Tsuji, Y. Osada, M. Kido, D. Inazu, Y. Hayashi, H. Tsushima, R. Hino, H. Fujimoto, Frontal wedge deformation near the source region of the 2011 Tohoku-Oki earthquake. Geophys. Res. Lett. 38, 2011GL048355 (2011).

[23] T. Nishimura, H. Munekane, H. Yarai, The 2011 off the Pacific coast of Tohoku earthquake and its aftershocks observed by GEONET. Earth Planets Space 63, 631–363 (2011).

[24] A. Hashima, T. W. Becker, A. M. Freed, H. Sato, D. A. Okaya, Coseismic deformation due to the 2011 Tohoku-oki earthquake: Influence of 3-D elastic structure around Japan. Earth Planets Space 68, 159 (2016).

[25] T. Iinuma, R. Hino, M. Kido, D. Inazu, Y. Osada, Y. Ito, M. Ohzono, H. Tsushima, S. Suzuki, H. Fujimoto, S. Miura, Coseismic slip distribution of the 2011 off the Pacific coast of Tohoku earthquake (M9.0) refined by means of seafloor geodetic data. J. Geophys. Res. Sol. Earth. 117, 2011GL048355 (2012).

[26] C. Kyriakopoulos, T. Masterlark, S. Stramondo, M. Chini, C. Bignami, Coseismic slip distribution for the Mw 9 2011 Tohoku-Oki earthquake derived from 3-D FE modeling. J. Geophys. Res.-Sol., Earth 118, 3837–3847 (2013).

[27] C. A. Williams, L. M. Wallace, The impact of realistic elastic properties on inversions of shallow subduction interface slow slip events using seafloor geodetic data. Geophys. Res. Lett. 45, 7462–7470 (2018).

[28] T. Sun, K. Wang, T. Fujiwara, S. Kodaira, J. He, Large fault slip peaking at trench in the 2011 Tohoku-oki earthquake. Nat. Commun. 8, 14044 (2017). 28074829 10.1038/ncomms14044 PMC5241695

[29] T. Fujiwara, C. Santos Ferreira, A. K. Bachmann, M. Strasser, G. Wefer, T. Sun, T. Kanamatsu, S. Kodaira, Seafloor displacement after the 2011 Tohoku-Oki earthquake in the northern Japan trench examined by repeated bathymetric surveys. Geophys. Res. Lett. 44, 11–833 (2017).

[30] S. Kodaira, T. Fujiwara, G. Fujie, Y. Nakamura, T. Kanamatsu, Large coseismic slip to the trench during the 2011 Tohoku-Oki earthquake. Annu. Rev. Earth Planet. Sci. 48, 321–343 (2020).

[31] T. Iwasaki, V. Levin, A. Nikulin, T. Iidaka, Constraints on the Moho in Japan and Kamchatka. Tectonophysics 609, 184–201 (2013).

[32] D. Zhao, W. Wei, Y. Nishizono, H. Inakura, Low-frequency earthquakes and tomography in western Japan: Insight into fluid and magmatic activity. J. Asian Earth Sci. 42, 1381–1393 (2011).

[33] M. Matsubara, T. Ishiyama, T. No, K. Uehira, M. Mochizuki, T. Kanazawa, N. Takahashi, S. Kamiya, Seismic velocity structure along the Sea of Japan with large events derived from seismic tomography for whole Japanese Islands including reflection survey data and NIED MOWLAS Hi-net and S-net data. Earth Planets Space 74, 171 (2022).

[34] D. Zhao, Z. Wang, N. Umino, A. Hasegawa, Mapping the mantle wedge and interplate thrust zone of the northeast Japan arc. Tectonophysics 467, 89–106 (2009).

[35] T. L. Grove, C. B. Till, E. Lev, N. Chatterjee, E. Médard, Kinematic variables and water transport control the formation and location of arc volcanoes. Nature 459, 694–697 (2009). 19494913 10.1038/nature08044

[36] I. Wada, M. D. Behn, Focusing of upward fluid migration beneath volcanic arcs: Effect of mineral grain size variation in the mantle wedge. Geochem. Geophys. Geosystems 16, 3905–3923 (2015).

[37] D. Zhao, Y. Katayama, G. Toyokuni, The Moho, slab and tomography of the East Japan forearc derived from seafloor S-net data. Tectonophysics 837, 229452 (2022).

[38] Y. Nakamura, S. Kodaira, B. J. Cook, T. Jeppson, T. Kasaya, Y. Yamamoto, Y. Hashimoto, M. Yamaguchi, K. Obana, G. Fujie, Seismic imaging and velocity structure around the JFAST drill site in the Japan Trench: Low Vp, high Vp/Vs in the transparent frontal prism. Earth Planets Space 66, 121 (2014).

[39] F. M. Chester, C. Rowe, K. Ujiie, J. Kirkpatrick, C. Regalla, F. Remitti, J. C. Moore, V. Toy, M. Wolfson-Schwehr, S. Bose, J. Kameda, J. J. Mori, E. E. Brodsky, N. Eguchi, S. Toczko; Expedition 343 and 343T Scientist, Structure and composition of the Plate-Boundary slip zone for the 2011 Tohoku-Oki earthquake. Science 342, 1208–1211 (2013). 24311682 10.1126/science.1243719

[40] Y. Hua, D. Zhao, G. Toyokuni, Y. Xu, Tomography of the source zone of the great 2011 Tohoku earthquake. Nat. Commun. 11, 1163 (2020). 32127532 10.1038/s41467-020-14745-8 PMC7054414

[41] L. Langer, H. N. Gharti, J. Tromp, Impact of topography and three-dimensional heterogeneity on coseismic deformation. Geophys. J. Int. 217, 866–878 (2019).

[42] F. Gallovič, W. Imperatori, P. M. Mai, Effects of three-dimensional crustal structure and smoothing constraint on earthquake slip inversions: Case study of the Mw 6.3 2009 L’Aquila earthquake: 2009 L’Aquila earthquake slip inversion. J. Geophys. Res. Solid Earth 120, 428–449 (2015).

[43] S. Tung, T. Masterlark, Coseismic slip distribution of the 2015 Mw 7.8 Gorkha, Nepal, earthquake from joint inversion of GPS and InSAR data for slip within a 3-D heterogeneous domain. J. Geophys. Res. Solid Earth 121, 2479–3503 (2016).

[44] J. Muto, B. Shibazaki, T. Iinuma, Y. Ito, Y. Ohta, S. Miura, Y. Nakai, Heterogeneous rheology controlled postseismic deformation of the 2011 Tohoku-Oki earthquake. Geophys. Res. Lett. 43, 4971–4978 (2016).

[45] A. Freed, A. Hashima, T. W. Becker, D. A. Okaya, H. Sato, Y. Hatanaka, Resolving depth-dependent subduction zone viscosity and afterslip from postseismic displacements following the 2011 Tohoku-oki, Japan earthquake. Earth Planet. Sci. Lett. 459, 279–290 (2017).

[46] S. Dhar, J. Muto, Y. Ohta, T. Iinuma, Heterogeneous rheology of Japan subduction zone revealed by postseismic deformation of the 2011 Tohoku-oki earthquake. Prog Earth Planet Sci 10, 9 (2023).

[47] R. Hino, Y. Ito, K. Suzuki, S. Suzuki, D. Inazu, T. Iinuma, Y. Ohta, H. Fujimoto, M. Shinohara, Y. Kaneda, Foreshocks and mainshock of the 2011 Tohoku Earthquake observed by ocean bottom seismic/geodetic monitoring, in American Geophysical Union, Fall Meeting 2011, Abstracts ID U51B-0008 (2011), vol. 2011, pp. 51–0008.

[48] T. Maeda, T. Furumura, S. Sakai, M. Shinohara, Significant tsunami observed at ocean-bottom pressure gauges during the 2011 off the Pacific coast of Tohoku Earthquake. Earth Planets Space 63, 803–808 (2011).

[49] J. Dong, W. Sun, X. Zhou, R. Wang, Effects of Earth’s layered structure, gravity and curvature on coseismic deformation. Geophys. J. Int. 199, 1442–1451 (2014).

[50] L. Langer, T. Ragon, A. Sladen, J. Tromp, Impact of topography on earthquake static slip estimates. Tectonophysics 791, 228566 (2020).

[51] L. Langer, T. Ragon, Accuracy of finite fault slip estimates in subduction zone regions with topographic Green’s functions and seafloor geodesy. J. Geophys. Res. Solid Earth 128, 1–16 (2023).

[52] C. Geuzaine, J. F. Remacle, Gmsh: A 3-D finite element mesh generator with built-in pre- and post-processing facilities. Int. J. Num. Meth. Eng. 79, 1309–1331 (2009).

[53] S. Puel, E. Khattatov, U. Villa, D. Liu, O. Ghattas, T. W. Becker, A mixed, unified forward/inverse framework for earthquake problems: Fault implementation and coseismic slip estimate. Geophys. J. Int. 230, 733–758 (2022).

[54] A. Logg, G. N. Wells, J. Hake, DOLFIN: A C++/Python finite element library, in *Automated Solution of Differential Equations by the Finite Element Method* (Springer, Berlin, Heidelberg, 2012), pp. 173–225.

[55] U. Villa, N. Petra, O. Ghattas, hIPPYlib: An extensible software framework for large-scale inverse problems. J. Open Source Softw. 3, 940 (2018).

[56] U. Villa, N. Petra, O. Ghattas, hIPPYlib: An extensible software framework for large-scale inverse problems governed by PDEs: Part 1: Deterministic inversion and linearized Bayesian inference. ACM Trans. Math. Software 47, 1–34 (2021).

[57] F. Tröltzsch, *Optimal Control of Partial Differential Equations: Theory, Methods, and Applications* (American Mathematical Soc., Washington, D.C., 2010), vol. 112.

[58] C. L. Lawson, R. J. Hanson, *Solving Least Squares Problems* (SIAM, Philadelphia, 1995).

[59] S. Ozawa, T. Nishimura, H. Suito, T. Kobayashi, M. Tobita, T. Imakiire, Coseismic and postseismic slip of the 2011 magnitude 9 Tohoku-Oki earthquake. Nature 475, 373–376 (2011). 21677648 10.1038/nature10227

[60] V. Akçelik, G. Biros, O. Ghattas, J. Hill, D. Keyes, B. Bloemen Waanders, Parallel algorithms for PDE-constrained optimization, in *Parallel Processing for Scientific Computing* (SIAM, 2006), pp. 291–322.

[61] P. Bird, An updated digital model of plate boundaries. Geochem. Geophys. Geosystems 4, 1027 (2003).

[62] G. Hayes, *Slab2–A Comprehensive Subduction Zone Geometry Model* (United States Geological Survey, 2018); 10.5066/F7PV6JNV. 30093602

[63] L. Siebert, T. Simkin, “Volcanoes of the world: An illustrated catalog of Holocene volcanoes and their eruptions” (Smithsonian Institution, Global Volcanism Program Digital Information Series GVP-3, 2002); volcano.si.edu/search_volcano.cfm.

