# Supplementary material for: Volcanic arc rigidity variations illuminated by coseismic deformation of the 2011 Tohoku-oki M9
Source: Sci Adv. 2024 Jun 5;10(23):eadl4264. doi: 10.1126/sciadv.adl4264 (PMC11152123; doi:10.1126/sciadv.adl4264)
Supplement: Supplementary file 1 — Figs. S1 to S10 Table S1 [file sciadv.adl4264_sm.pdf]

Science Advances
AAAS

Supplementary Materials for

Volcanic arc rigidity variations illuminated by coseismic deformation of the 2011 Tohoku-oki M9

Simone Puel *et al.*

Corresponding author: Simone Puel, spuel@utexas.edu

Sci. Adv. **10**, eadl4264 (2024)
DOI: 10.1126/sciadv.adl4264

This PDF file includes:

Figs. S1 to S10
Table S1

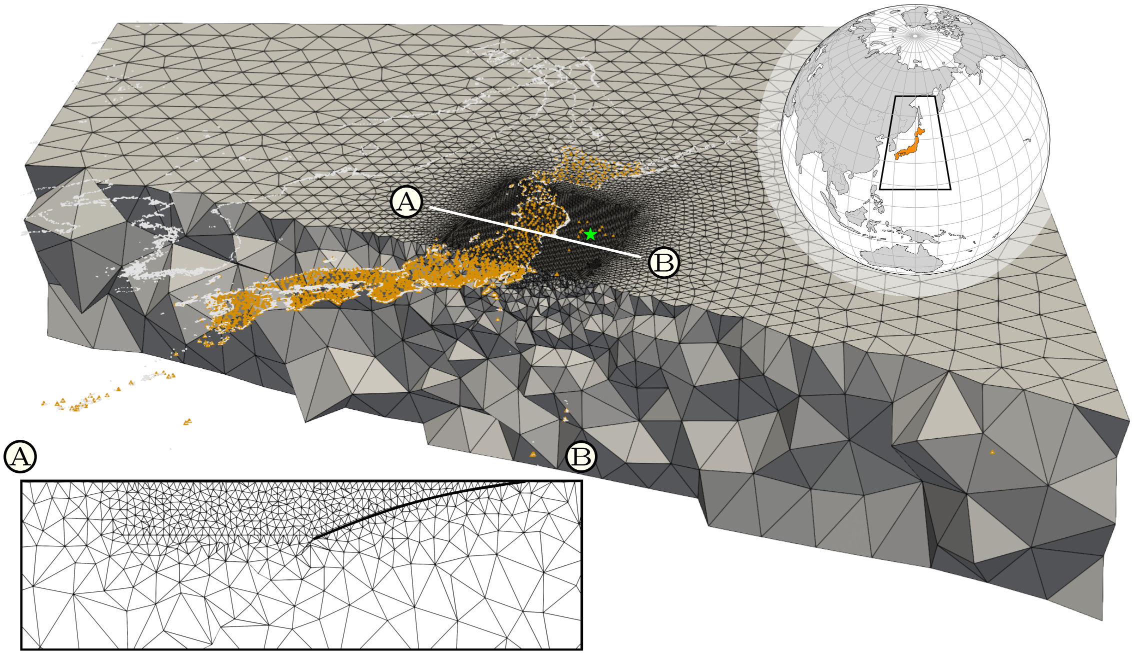

Fig. S1. 3-D finite-element mesh of Honshu region, Japan, illustrating the spatial discretization of the computational mesh. Orange triangles indicate the location of the 1,296 geodetic sensors used in this study, including 13 offshore stations. The green star denotes the epicenter of the Tohoku-oki earthquake. Profile A-B provides a detailed view of the fault and domain discretization, with the smallest tetrahedra having a characteristic length of ≈10 km.

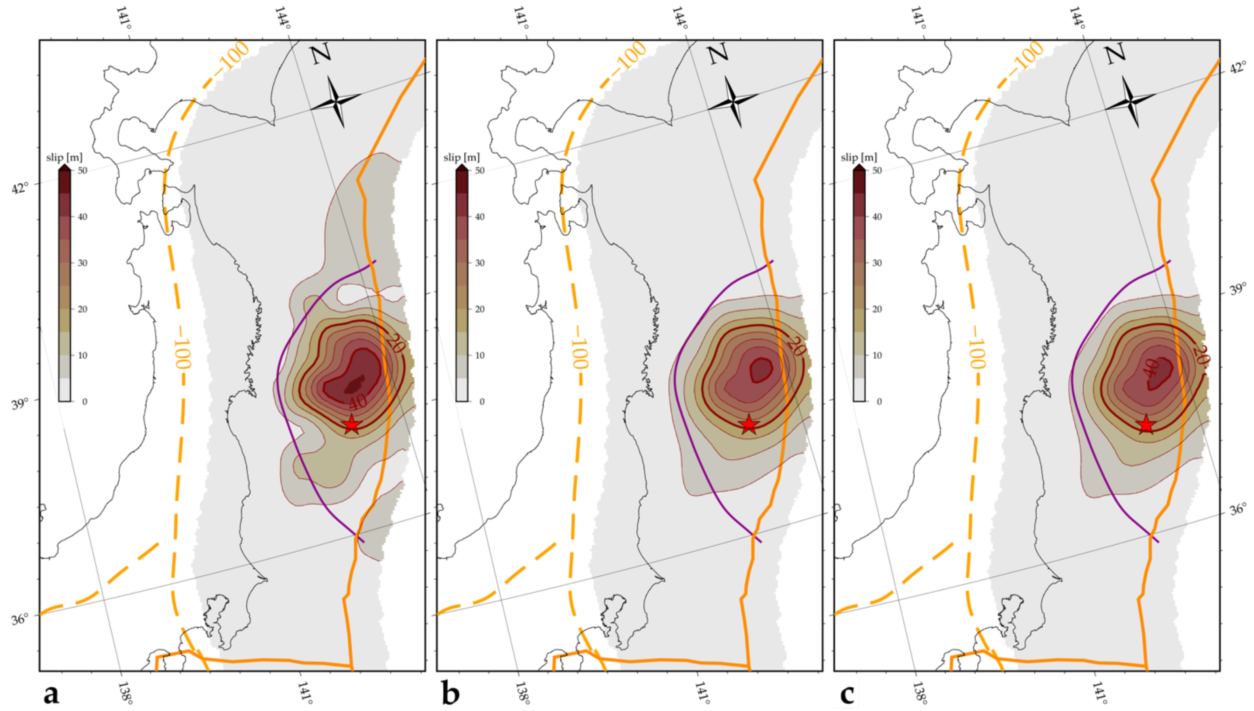

Fig. S2. Comparison of inferred coseismic slip distributions between homogeneous and heterogeneous structures, including a 10° steeper reference slab geometry. (**a**) Estimated fault slip distribution for a homogeneous medium slip inversion, and for a joint inversion for fault slip and 3-D shear modulus variations (**b**). (**c**) Same as (**b**) but with a 10° deeper slab geometry for the Pacific plate. The dark orange lines denote major plate boundaries (25), and the orange dashed line depicts the 100 km contour line of the subducting Pacific slab (60). The red star indicates the Tohoku-oki epicenter, and the purple line represents the 5 m slip contour from a previous coseismic inversion (24).

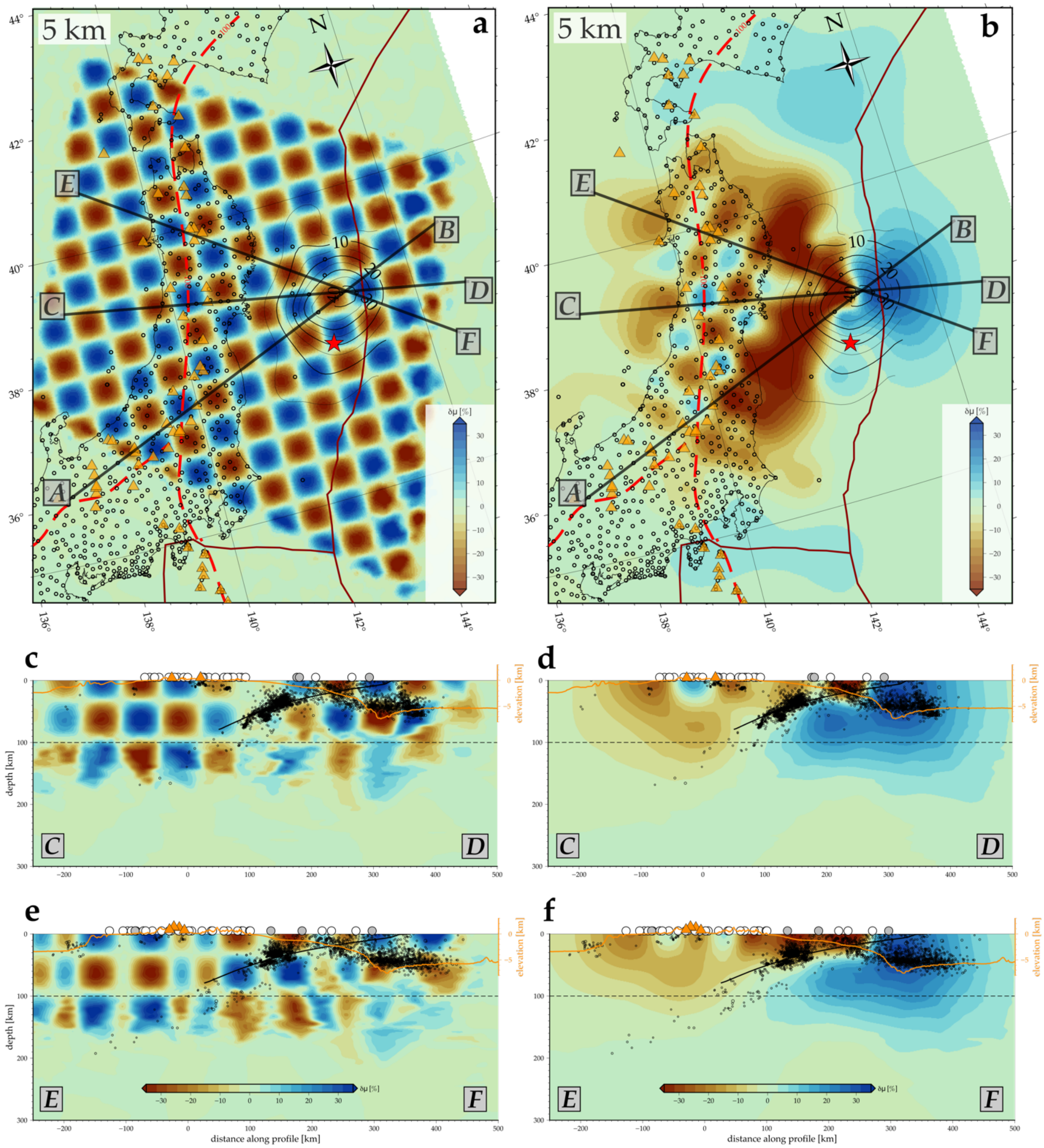

Fig. S3. Results of checkerboard resolution test. Figures show the recovery of a sinusoidal rigidity perturbation of 50 km × 50 km × 50 km size with a maximum amplitude of ±35%. (**a**-**b**) Map view of checkerboard input and the corresponding recovery at a depth of 5 km, respectively. The input slip distribution is obtained from homogeneous slip inversion, while the

recovery is shown with 5 m black contours. Plate boundaries, slab contours, and earthquake epicenter as in Fig. 2. Vertical profiles (**c**-**e**) and (**d**-**f**) depict the input and recovered resolution tests, respectively. Topographic elevation, volcano and station locations, and seismicity as in Fig. 3.

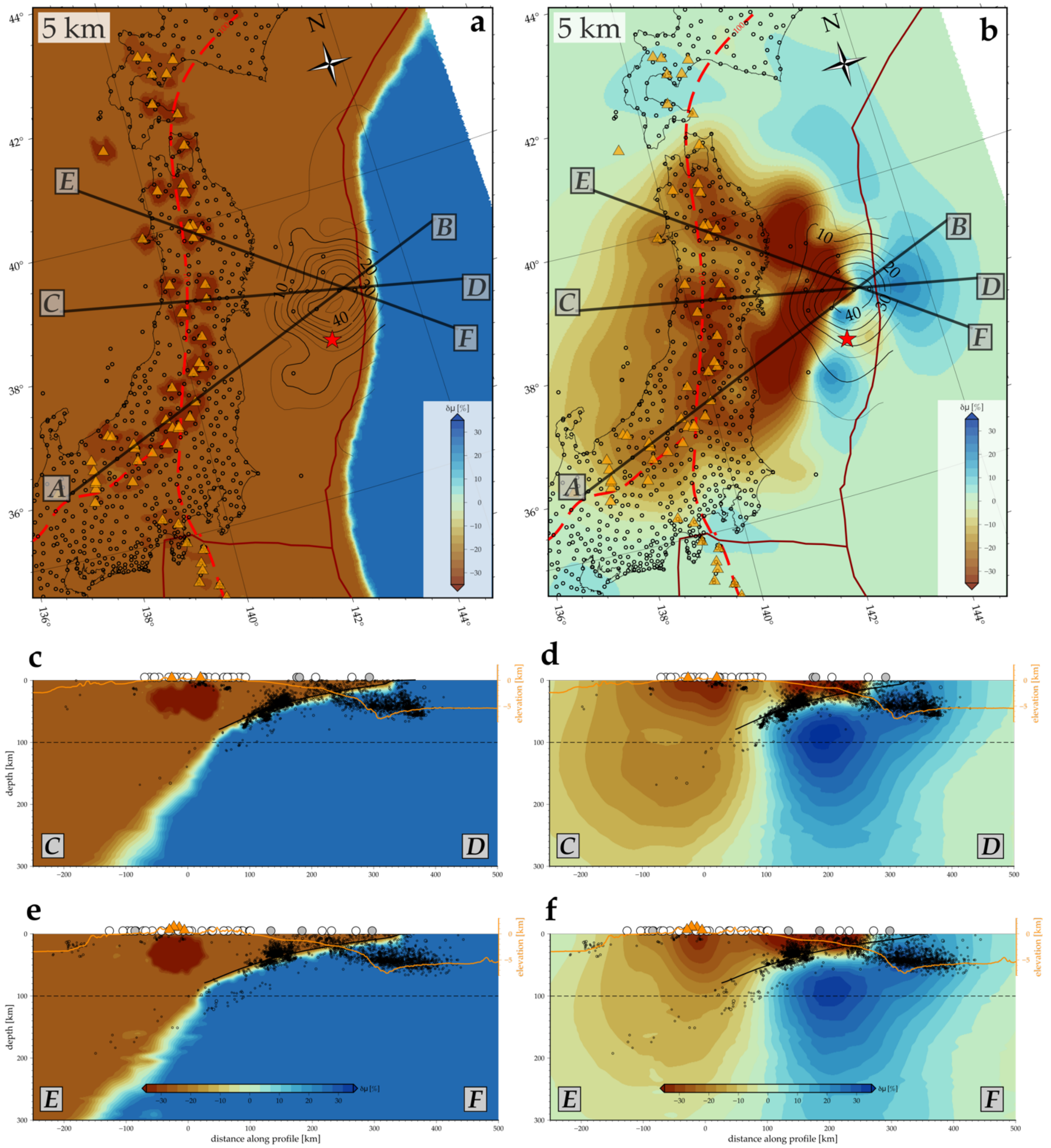

Fig. S4. Results from 3-D recovery tests for a synthetic subduction zone structure. (**a**) Horizontal slice of the input structure at a depth of 5 km, illustrating a subducting slab with higher rigidity (75 GPa), weaker overriding plate (45 GPa), and spherical anomalies beneath the volcanic arc (35 GPa) with a radius of 30 km, with 60 GPa background shear modulus. The input

slip distribution from homogeneous slip inversion is represented by black contours with 5 m intervals (Fig. 1b). (**b**) Recovered slip distribution (black contours) and rigidity structure at a depth of 5 km. Plate boundaries, slab contours, and earthquake epicenter as in Fig. 2. (**c**-**e**) and (**d**-**f**) display the input and recovered vertical profiles of sections C-D and E-F, respectively. Topographic elevation, volcano and station locations, and seismicity as in Fig. 3.

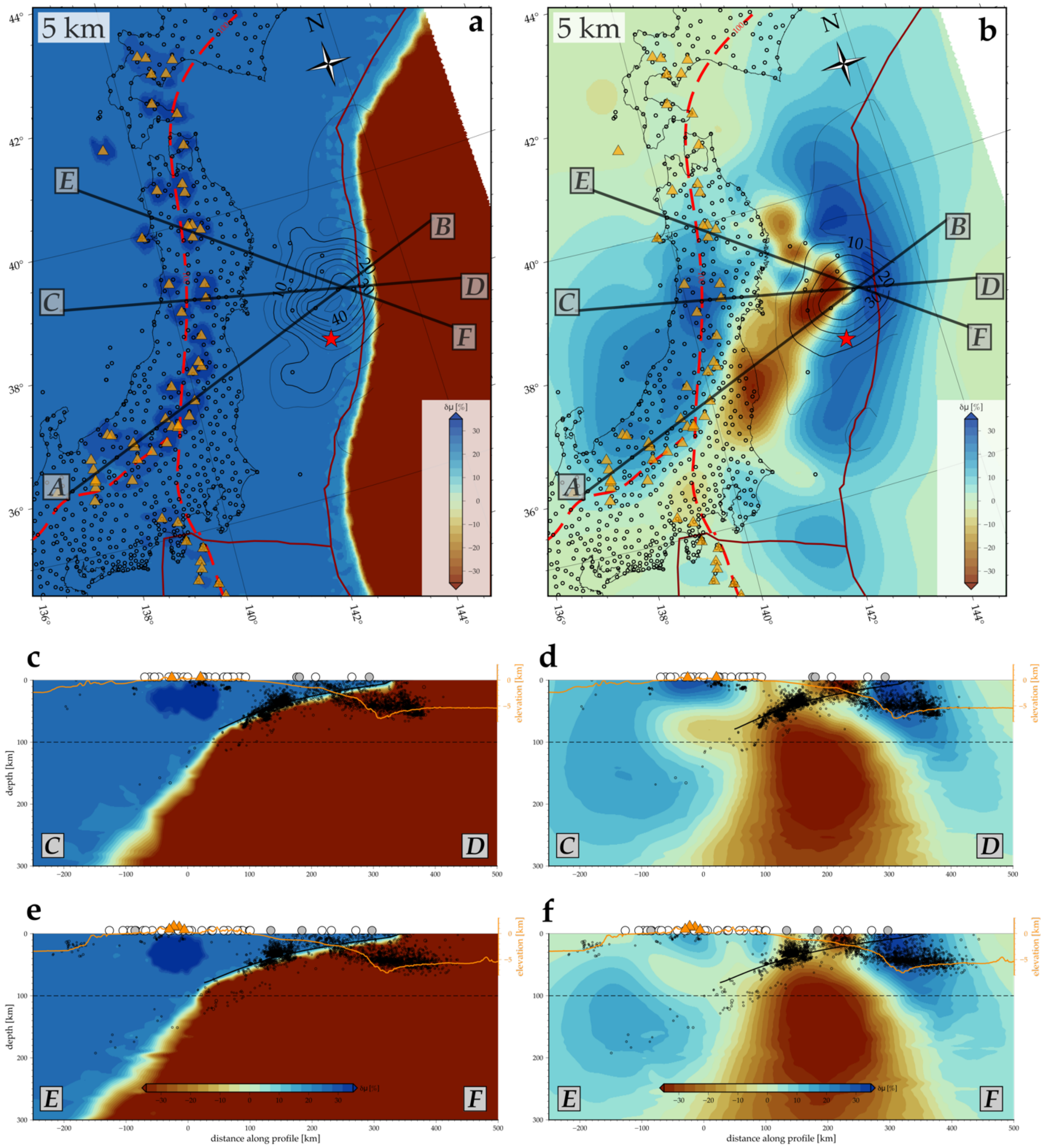

Fig. S5. Results from recovery tests for a hypothetical, antithetical synthetic subduction zone structure with a weak slab. (**a**) Horizontal slice of the input structure at a depth of 5 km, illustrating a subducting slab with lower rigidity (35 GPa), a stronger overriding plate (75 GPa), and spherical anomalies beneath the volcanic arc (85 GPa) with a radius of 30 km. The input slip

distribution from homogeneous slip inversion is represented by black contours with 5 m intervals (Fig. 1b). (**b**) Recovered slip distribution (black contours) and rigidity structure at a depth of 5 km. Plate boundaries, slab contours, and earthquake epicenter as in Fig. 2. (**c-e**) and (**d-f**) display the input and recovered vertical profiles of sections C-D and E-F, respectively. Topographic elevation, volcano and station locations, and seismicity as in Fig. 3.

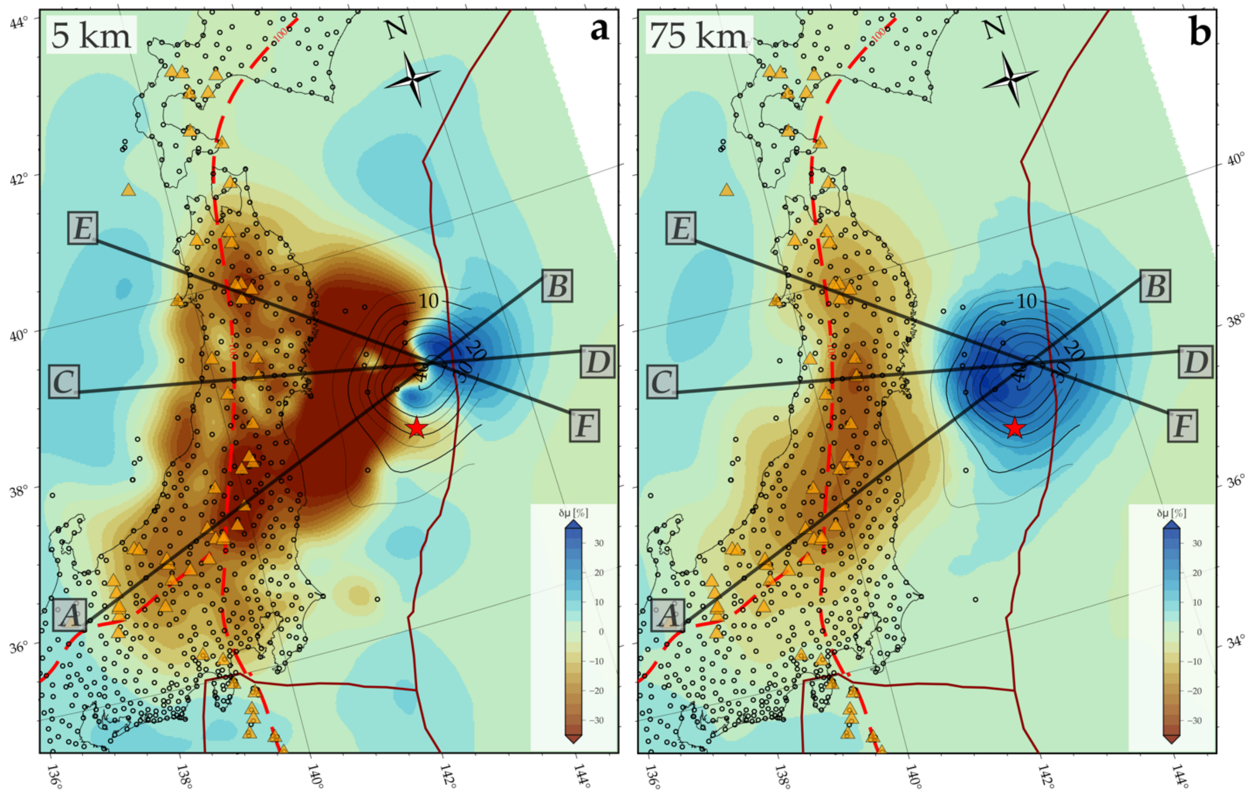

Fig. S6. Map view of rigidity variations from joint inversion with a 10° steeper than reference slab geometry. Horizontal slices illustrating the rigidity variations at 5 km (**a**) and 75 km (**b**). Plate boundaries, slab contours, and earthquake epicenter as in Fig. 2. Black slip contour matches that of Fig. S2c. The three vertical profiles in Fig. S7 are represented by black lines.

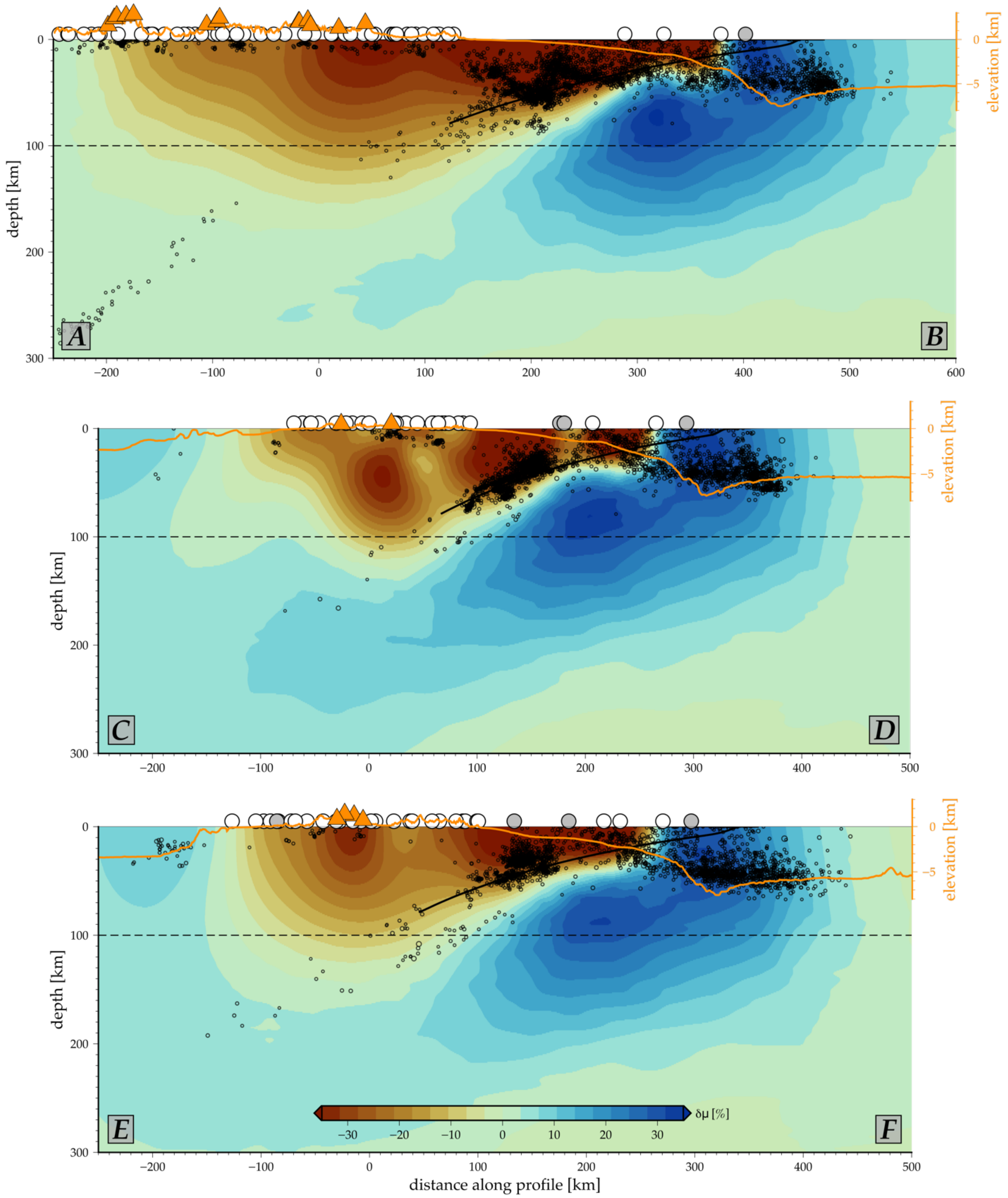

Fig. S7. Vertical profiles of rigidity variations from joint inversion with a 10° steeper slab geometry. The solid black line indicates the 10° deeper slab geometry for the Pacific plate. The

corresponding horizontal traces are illustrated in Fig. S6. Topographic elevation, volcano and station locations, and seismicity as in Fig. 3.

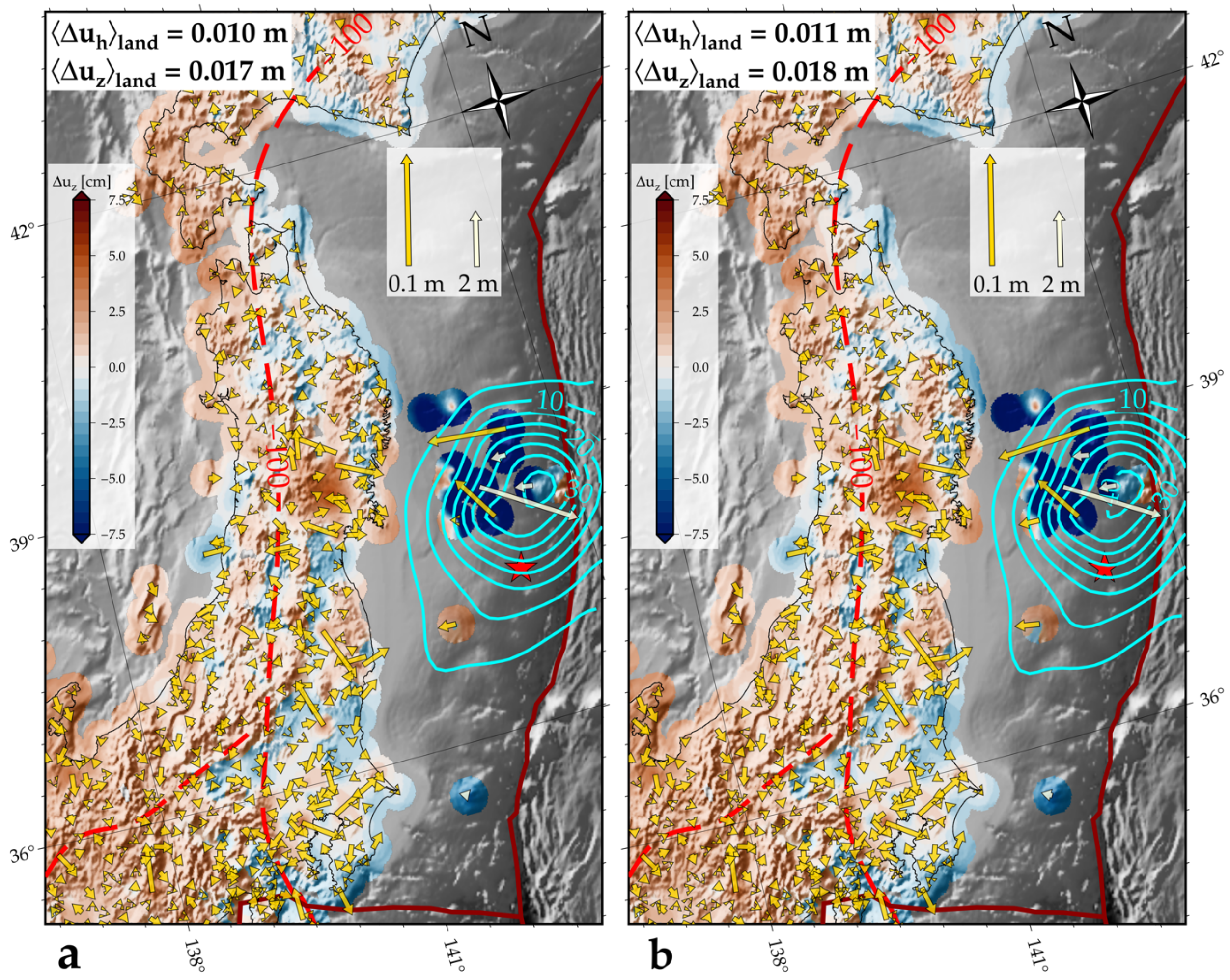

Fig. S8. Comparison of residuals for reference joint inversion and joint inversion with a 10° steeper slab. (**a**) Reference joint inversion of Fig. 1c and, (**b**), joint inversion incorporating a 10° steeper slab geometry of the Pacific plate. Plate boundaries, slab contours, and earthquake epicenter as in Fig. 1. Cyan slip contours correspond to panels (b) and (c) in Fig. S2. Brackets indicate the root-mean-square (RMS) residuals of horizontal ($\boldsymbol{u_h}$) and vertical ($\boldsymbol{u_z}$) displacements, restricted to the map area and on land.

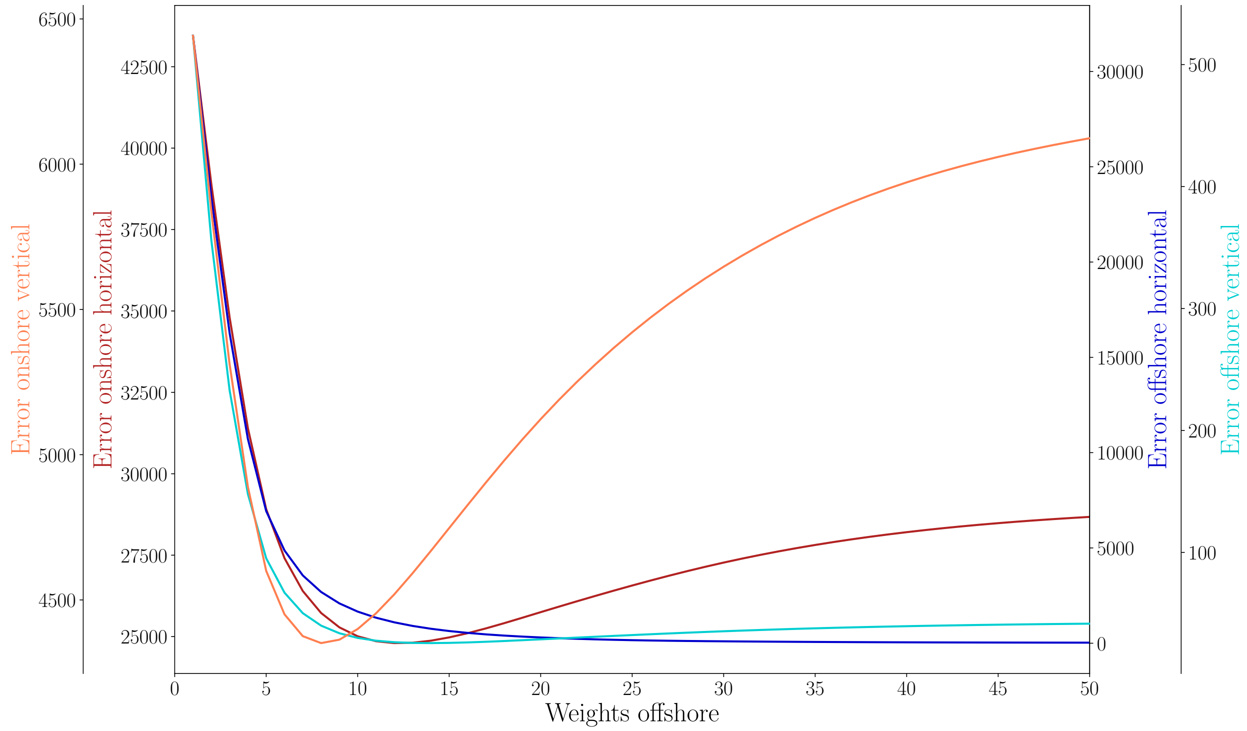

Fig. S9. L_2-norm error of residuals for data weights. L_2-norm error of residuals between the observed and predicted surface displacements from coseismic slip inversions assuming a homogeneous subduction zone structure. The misfits are weighted by the noise uncertainties. The weighting ratios were selected by minimizing the error, identifying the intersection of the different curves. We obtained a preferred ratio of $\mathbf{1}:\frac{\mathbf{1}}{\mathbf{2}}:\frac{\mathbf{1}}{\mathbf{13}}:\frac{\mathbf{1}}{\mathbf{10}}$ for the horizontal components of the onshore data, vertical components of the onshore data, seafloor acoustic data, and pressure gauges vertical displacements, respectively.

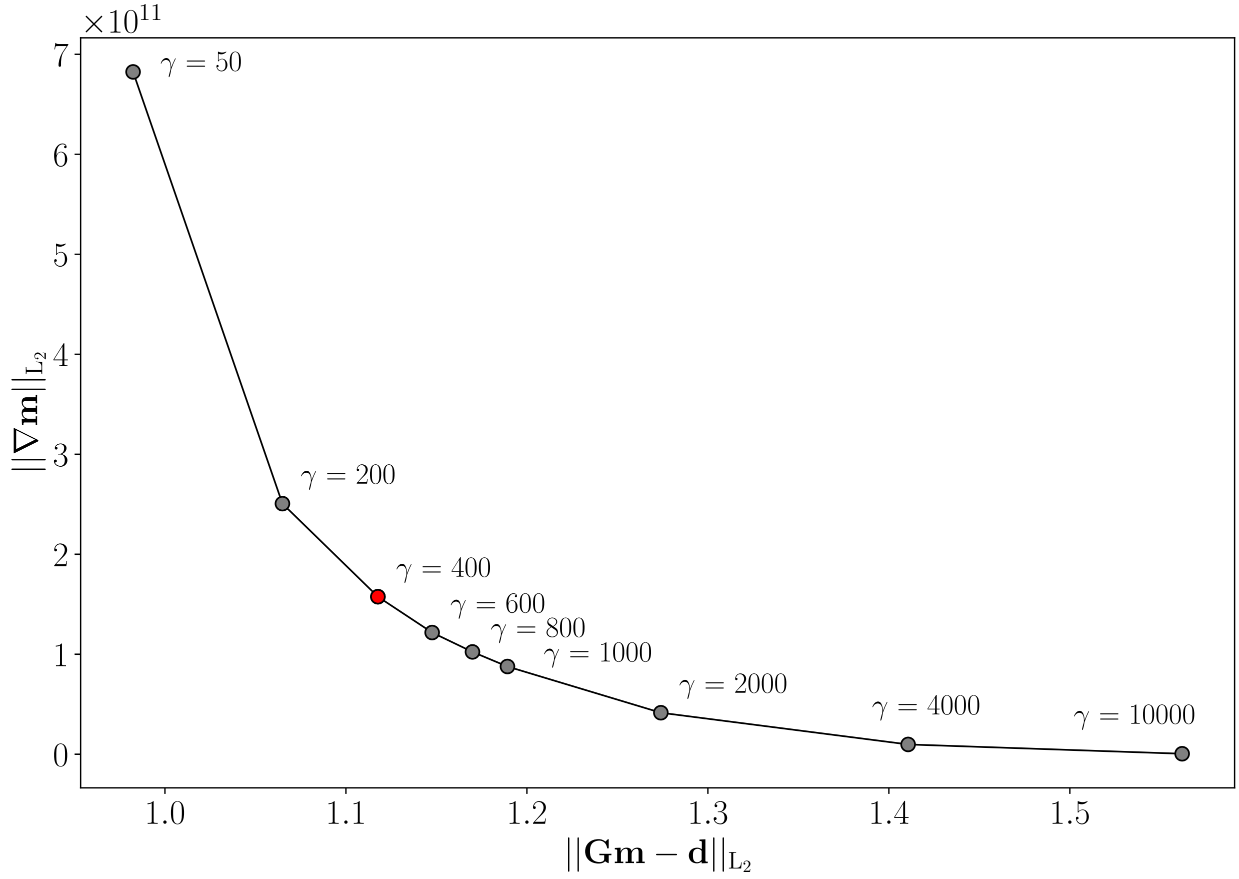

Fig. S10. L-curve for selection of damping parameters in the joint inversion. L-curve (53) illustrating the relationship between data and model misfit, aiding in the determination of the best damping parameter $\boldsymbol{\gamma}$. The gray dots represent joint inversion models with varying $\boldsymbol{\gamma}$ values, while the red dot indicates the preferred value of 400.

Station code	Type of station	Location	σ_h [m]	σ_z [m]	Reference
-	GPS	on land	0.004	0.015	ref. (54)
GJT3	GPS/A	offshore	0.5	0.012	ref. (62)
GJT4	GPS/A	offshore	0.5	N/A	ref. (21)
MYGI	GPS/A	offshore	0.1	0.1	ref. (62)
MYGW	GPS/A	offshore	0.1	0.1	ref. (20)
FUKU	GPS/A	offshore	0.1	0.1	ref. (62)
KAMS	GPS/A	offshore	0.5	0.5	ref. (62)
KAMN	GPS/A	offshore	0.5	0.5	ref. (20)
CHOS	GPS/A	offshore	0.5	0.5	ref. (20)
TJT1	APG	offshore	-	0.014	ref. (22)
P02	APG	offshore	-	0.016	ref. (45)
P06	APG	offshore	-	0.015	ref. (45)
TM1	APG	offshore	-	0.2	ref. (46)
TM2	APG	offshore	-	0.2	ref. (46)

Table S1. Observational errors in the coseismic surface geodetic data of the 2011 M9 Tohoku-oki earthquake in Japan. The first and second columns display the station name and the type of sensor used (GPS, GPS/A, or APG). The third column specifies the location of the observations, whether on land or offshore. The fourth and fifth columns represent the standard deviations of the data noise in meters for the horizontal components (north and east) and the vertical component, respectively. The last column denotes the reference source for the observational errors. APG sensors exclusively provide vertical displacement measurements.
